# Analysis of Prediabetes in Veterans: Assessing Diagnosis and Population Characteristics Across a Region of Veterans Affairs Health Care Systems

**DOI:** 10.3390/healthcare13233061

**Published:** 2025-11-26

**Authors:** Beth D. Greck, Amanda T. Pons-Junkins, Robert S. Colling, Paula Richley Geigle

**Affiliations:** 1 Western North Carolina VA Health Care System, Asheville, NC 28805, USA; robert.colling@va.gov (R.S.C.); geigle@comcast.net (P.R.G.); 2 Department of Epidemiology and Prevention, Wake Forest University School of Medicine, Winston-Salem, NC 27101, USA; amanda.ponsjunkins@advocatehealth.org

**Keywords:** prediabetes diagnosis, veterans, hemoglobin A1c

## Abstract

**Background/Objectives**: Currently, 873,000 patients within the VA Health Administration potentially have prediabetes, based on HbA1c. The primary objective of this analysis was to describe and compare Veterans with and without a documented prediabetes diagnosis, across age, sex, race, rurality, and select comorbidities. Secondary objectives included the following: (1) Identify prediabetes diagnosis documentation trends between the VISN 6 facility complexity and (2) evaluate the relationship between specific prescribed medication classes for Veterans and a documented prediabetes diagnosis. **Methods**: Veterans presenting to a VISN 6 VA facility within the past 24 months for at least one visit were identified. Veterans were excluded if they had a documented diabetes diagnosis, an HbA1c > 6.5%, or missing demographic data. Veterans with an HbA1c >/= 5.7% to 6.4% were noted if they had a documented prediabetes diagnosis. Data was analyzed between groups (diagnosis code vs. no diagnosis code) using descriptive analyses not designed for causal inference. **Results**: Of the 105,737 Veterans meeting the analysis criteria, over 68% did not have a documented diagnosis. Median age was similar between groups. Black and female Veterans were more likely to have a documented prediabetes diagnosis. Documentation among VISN 6 facilities varied (nearly 28% to over 36%). Those with dyslipidemia, hypertension, or heart failure were less likely to have a documented diagnosis, as well as those with depressive, anxiety, and stress and adjustment disorder diagnoses. Those prescribed metformin or a GLP1 agonist had a higher rate of diagnosis documentation, while over 71% of Veterans prescribed an antipsychotic had no documented diagnosis. There was no linear trend with documentation across facility complexity or rurality. **Conclusions**: Most VISN 6 Veterans with prediabetes do not have a documented diagnosis. There is an opportunity to increase provider awareness of prediabetes diagnosis documentation, particularly among at-risk Veterans, to improve type 2 diabetes prevention in Veterans.

## 1. Introduction

The Center for Disease Control (CDC) reported that 38% of all adults in the United States had prediabetes between 2017 and 2020 [1]. Prediabetes, also called impaired glucose tolerance, impaired fasting glucose, or intermediate hyperglycemia, progresses to type 2 diabetes at an annualized rate of 5–10% [2]. The Veteran population displays a higher diabetes prevalence compared to the general population with nearly 25% of Veterans in the health care system with a diabetes diagnosis. National VA data demonstrates that there may also be a higher prevalence of prediabetes in Veterans. There are over 1.7 million Veterans within VHA, nearly 119,000 in the VA Mid-Atlantic Health Care Network alone (VISN 6), who potentially have prediabetes, based on their HbA1c result [3].

It is important to identify and characterize the current Veteran population with prediabetes, not only because of the existing high prevalence of type 2 diabetes, but because the Veteran population holds unique risk factors for developing prediabetes and type 2 diabetes. Obesity is a known risk factor for diabetes and prediabetes [4]. According to Breland et al., obesity rates range from 28% to 49% among VHA facilities, in comparison to the nearly 42% of US adults who are obese [5,6]. Veterans also experience a higher prevalence of serious mental illness. Liu et al. found that 9.6% Veterans were diagnosed with depression, higher than the 6.7% reported by adults in the United States [7]. Serious mental illness, such as major depressive disorder and schizophrenia, is associated with a greater risk of type 2 diabetes in Veterans compared to Veterans without serious mental illness [8]. Not only can serious mental illness increase the risk of diabetes, but so can the medications often prescribed to treat these conditions. There is well-documented evidence of these medications, such as atypical antipsychotics, contributing to metabolic syndrome, including hyperglycemia, weight gain, and hypertension [9].

Identifying Veterans with prediabetes is crucial to type 2 diabetes prevention and its complications. A relationship exists between prediabetes and the micro- and macrovascular complications often associated with diabetes [10]. A meta-analysis from 2021 found that prediabetes is associated with a risk of all-cause mortality [11]. A cross-sectional study from Ishikawa et al. reported an over 39% prevalence of prediabetes in people with heart failure [12]. With these costly potential complications, investigating prediabetes diagnosis patterns in VISN 6 Veterans with cardiovascular risk factors is critical. Dyslipidemia, a condition associated with cardiovascular disease, is a known risk factor for type 2 diabetes (Von Eckardstein and Sibler 2011; Davis et al. 2018 [13,14]). A retrospective study including six VA medical facilities identified that over 36% of Veterans had dyslipidemia [15]. Hypertension, another risk factor for type 2 diabetes and cardiovascular disease, affects nearly 45% of Veterans in the VHA [15,16]. Heart failure and prediabetes have a bidirectional relationship, which demonstrates the importance of assessing prediabetes status for the prevention and management of heart failure [17,18,19]. With such a prevalence of these risk factors among Veterans, routine screening and documentation of prediabetes is indicated and necessary for the prevention of diabetes and cardiovascular disease.

Veterans are more likely reside in rural areas than the general population. Living in rural areas brings its own challenges for health care and disease management [20,21]. Rural populations tend to have a higher prevalence of diabetes and risk factors for diabetes, as well as a higher diabetes-related mortality [22]. While there is limited data regarding prediabetes prevalence among Veterans in rural areas, based on diabetes prevalence it is likely greater than in urban areas, making this a key at-risk population for prediabetes.

The purpose of this retrospective analysis is to characterize the Veteran population within the VISN 6 network with and without a documented prediabetes diagnosis. We assess various known risk factors for prediabetes and diabetes to guide better population screening and education for prediabetes. This study provides insight into the prediabetes population across VISN 6, including diagnosis patterns, and provides directions for improvements in addressing prediabetes in Veterans.

## 2. Materials and Methods

### 2.1. Study Design and Patient Population

This analysis is a cross-sectional, observational, retrospective review of patient data available from North Carolina and Virginia VA Medical Centers (VAMCs) included in the Mid-Atlantic Health Care Network (VISN 6) to understand factors that may influence a formal diagnosis of prediabetes within the system.

Data was retrieved from the on-premises cloud server farm, the Corporate Data Warehouse (CDW; Austin, TX, USA), using T-SQL (SQL Server Management Studio 0.2.37.0, Redmond, WA, USA) for living Veterans aged 35–89 with at least one outpatient encounter at a VISN 6 VA Medical Center (VAMC) within the previous 24 months (24 June 2023 through 23 June 2025). VISN 6, also known as the Mid-Atlantic Health Care Network, encompasses VAMCs in North Carolina and Virginia. Duplicate encounters were excluded from the initial query. Patients were included in the analysis if they had a qualifying lab for prediabetes from 24 June 2023 to 23 June 2025. Prediabetes was defined as an HbA1c result equal to or greater than 5.7% and less than 6.5% in the 24-month analysis window from both inpatient and outpatient labs. Because it is VHA policy that patients do not fast for blood work, this analysis did not include fasting plasma glucose in inclusion criteria. Patients were excluded if they were previously diagnosed with diabetes with ICD-10 codes E10.xx, E11.xx, or E13.xx.

### 2.2. Outcome Variable

The outcome variable was defined as the presence of a diagnosis of prediabetes through at least one documented instance of ICD-10 code R73.xx, which encompasses impaired fasting glucose, impaired glucose tolerance, and prediabetes.

### 2.3. Exposure Variables

Patient race, age, and sex were included in the analysis to observe differences in sociodemographic characteristics between patients with and without a documented diagnosis of prediabetes. Within each group, we used ICD-10 codes to identify patients with medical conditions associated with prediabetes and diabetes risk, including hypertension (I10), dyslipidemia (E78), and heart failure (I50). Because Veterans have a higher prevalence of serious mental health conditions associated with diabetes risk, we included ICD-10 codes for depression and major depression (F32, F33), anxiety disorders including PTSD (F41, F43), and schizophrenia (F20). Common medication classes with a known risk for diabetes or risk factors were included in the analysis. Prescriptions were flagged as yes/no if a patient had an active or suspended prescription for the following medication classes: antipsychotics (CN701, CN709), prednisone (HS051) with a written quantity of 90 or greater, immunosuppressants (IM599, IM600), GLP1 agonists (HS509), weight loss medications (GA751, GA900), and metformin (HS502).

To assess rurality, addresses were coded as Urban, Rural, Highly Rural, and Insular (U, R, H, I) based on VHA designation determined by the census tract in which the Veteran resided. To understand differences in the size and capabilities of different VAMCs on diagnosis patterns of prediabetes, we included VA facility complexity in the analysis. Complexity level reflects the volume, patient risk level, clinical programs, and size of clinical and teaching programs of the VA facility. All VISN 6 Medical Centers are designated as complexity 1, further designated as 1a, 1b, or 1c, with ‘1a’ being the most complex.

### 2.4. Statistical Analysis

**Figure 1:**
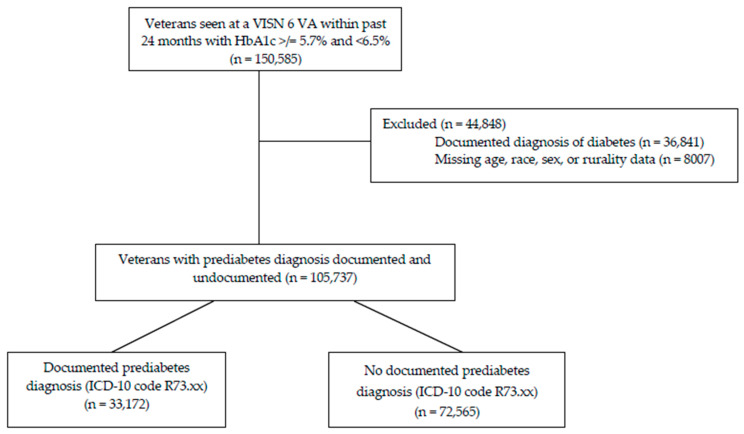
Consort diagram.

Data cleaning and analysis was performed in SAS 9.4 (SAS Institute, Cary, NC, USA). Patients with a diagnosis of diabetes or missing information on age, sex, or race were removed from the analysis by listwise deletion (Figure 1). After removal of those with an ICD-10 code for diabetes in the last two years (n = 36,841) or with missing demographic data (n = 8007), remaining patients with a qualifying HbA1c for prediabetes (n = 105,737) were categorized by presence of a documented diagnosis of prediabetes (R73.xx). Descriptive statistics were reported for demographics (Table 1), active medication prescriptions identified for analysis (Table 2), and the conditions associated with diabetes risk and prediabetes (Table 3). Bivariate analysis was performed on patients with a documented prediabetes diagnosis code versus patients without a documented code. Bivariate analysis was performed by using the Kruskal–Wallis test for continuous variables (age) and the Chi-Square test of independence for categorical variables (gender, race, prescriptions, and risk factor ICD-10 codes). Statistical significance was set at α = 0.05, and no corrections for multiple comparisons were applied. Table 4 shows prediabetes documentation by facility complexity (1a, 1b, or 1c).

### 2.5. Ethical Considerations

This study was reviewed by the WNCVAHCS Institutional Review Board and deemed not to be research.

## 3. Results

### 3.1. Diagnosis and Demographics

Baseline demographics are displayed in Table 1. The average age (median 62 years) for those with a prediabetes diagnosis and those without a diagnosis was similar. Both groups were predominantly male (84%), mirroring VHA enrollees nationally. Within VISN 6, female patients were shown to have a higher proportion of documented prediabetes than undocumented prediabetes (18.7% versus 15.2%), unlike male patients. Black patients had a higher proportion of documented prediabetes versus non-documented prediabetes (45.5% vs. 38.4%), while white patients had a lower proportion of coded diagnosis (47.7% vs. 54.3%) within the study sample.

### 3.2. Medications

Table 2 summarizes the findings among the cohorts prescribed specific medication classes. While only a small percentage of Veterans were prescribed an antipsychotic, there is an established association between hyperglycemia and antipsychotic medications. Despite this knowledge, over 71% of Veterans with prediabetes prescribed an antipsychotic displayed no documented prediabetes diagnosis. This was similar among Veterans prescribed immunosuppressants and chronic prednisone.

Obesity is a well-recognized risk factor for prediabetes, yet only 32.4% of Veterans prescribed a weight loss medication had a documented prediabetes diagnosis. Metformin demonstrated potential benefit in prediabetes through the Diabetes Prevention Program study yet just 54% of Veterans prescribed metformin had a documented diagnosis.

### 3.3. Concurrent Medical Conditions

Table 3 captures the incidence of concurrent medical conditions in each prediabetes group. Cardiovascular conditions associated with hyperglycemia were evaluated. Veterans with a lipid disorder or hypertension were less likely to have a documented prediabetes diagnosis. The lack of documentation was even higher, at over 30%, for those with heart failure.

Mental illness comorbidities, including schizophrenia, depressive and major depressive disorders, anxiety disorders, and stress and adjustment disorders, were included in our evaluation. Schizophrenia represented a very small percentage of the population (<1%), but the majority with prediabetes did not have a documented diagnosis. The lack of documented diagnosis was similar, around 67%, in Veterans with depressive, anxiety, and stress and adjustment disorder diagnoses.

### 3.4. Comparison of VISN Facilities

The percentage of Veterans with a documented diagnosis was similar across the seven facilities. Documentation ranged from 27.9% to 36.3% between facilities, with an average of 31.4%. When facility complexity was considered, the percentage of Veterans without a diagnosis was similar, apart from level 1b facilities. This complexity level, which also had the smallest prediabetes population, demonstrated a lower documentation rate (Table 4). When assessing rurality, interestingly, urban and highly rural Veterans had similar rates of diagnosis, while rural Veterans experienced a lower rate of documented diagnosis (Table 5).

## 4. Discussion

Almost 69% of Veterans meeting the criteria did not have a documented diagnosis of prediabetes. A study in 2022 found a similar gap in prediabetes diagnosis within the active military population. They found that less than half of those meeting the criteria for prediabetes actually had a documented diagnosis [23].

Much of the analyzed population were white males. This is reflective of the general VHA population and may not be applicable to the general population. Though the population numbers of Black Veterans and female Veterans were small, they were more likely to have a documented prediabetes diagnosis, and this should be further explored.

We analyzed certain concurrent health conditions associated with prediabetes and type 2 diabetes. We found that Veterans with cardiovascular conditions including dyslipidemia, hypertension, and heart failure were less likely to have a documented diagnosis. These are known risk factors for type 2 diabetes, and therefore we would anticipate a higher rate of prediabetes recognition through documentation. There were similar rates of diagnosis documentation among Veterans with depressive, anxiety, and stress and adjustment disorder diagnoses, but this rate was low. These conditions can increase the risk of diabetes due to their association with obesity and medications that may lead to hyperglycemia.

The assessment of medications that may impact glucose found a higher rate of diagnosis documentation in those prescribed metformin or a GLP1 agonist. This was expected based on evidence that these medication classes positively impact glucose and weight. Despite the known hyperglycemia risk, over 71% of Veterans with prediabetes prescribed an antipsychotic displayed no documented diagnosis. There was a small volume of prescriptions for immunosuppressants and chronic prednisone, but the lack of a documented diagnosis was similar to those prescribed antipsychotics.

Documentation rates among VISN 6 facilities based on facility complexity indicated that a lack of documentation occurred at a range of VAs. More detailed assessment may reveal potential contributors to the lower documentation rate among 1b facilities. Regarding rurality, the lack of an ordered trend from highly rural, to rural, to urban needs further investigation, including a multivariate analysis to account for variables such as those gathered in this study.

Limitations exist with this retrospective analysis. It is possible that a Veteran may be informed of a prediabetes diagnosis, but documentation was not completed by the provider. Documentation of ICD-10 codes may differ based on the provider or facility, or may be documented externally for Veterans seeking care outside of the VA system. These findings are limited to the facilities within this geographic area and may not reflect the VA as a whole or non-VA facilities. Because the focus of this evaluation was an exploratory evaluation of the VISN 6 geographic region, this study is primarily descriptive. Because this study observes clinical characteristics, demographic data, and a diagnosis of prediabetes over at any time over a two-year cross-sectional period, multivariate regression cannot be used for causal analysis, as comorbid diagnoses and related prescriptions are not specified to occur prior to a diagnosis of prediabetes. Moreover, prescriptions for prediabetes treatment, such as metformin, are often associated with a formal diagnosis, indicating that perhaps documentation of the diagnosis is overlooked. Providers may have varying experience and understanding about prediabetes, contributing to the low frequency of diagnosis documentation. Additionally, we acknowledge HbA1c testing limitations in terms of the screening and diagnosis of prediabetes. Various medical conditions impacting hemoglobin concentrations or red blood cell turnover could potentially lead to unreliable HbA1c measurements. Some evidence indicates that certain racial groups may experience higher baseline HbA1c levels, which could lead to the misdiagnosis of some patient populations. VHA clinical guidance for prediabetes diagnosis criteria includes HbA1c ≥ 5.7–6.4% and fasting plasma glucose (FPG) ≥ 100 mg/dL (5.5 mmol/L) [24]. We did not include FPG in our review, as no retrospective method was available to confirm a Veteran’s fasting state when the blood was collected. VHA policy states that labs do not require fasting unless specifically stated or requested by the ordering provider [25].

While this was an exploratory analysis, future research could include multivariate analysis to assess the impact of these diabetes risk factors that are more common among the Veteran population. Our findings may also guide qualitative research assessing provider education and understanding of prediabetes, including the clinical impact of the condition and evidenced-based interventions that can prevent progression to type 2 diabetes.

## 5. Conclusions

The risks associated with prediabetes are well documented, including not only potential progression to type 2 diabetes but also complications such as cardiovascular disease. Despite the known risks, there is an apparent gap in diagnosis documentation. This missing documentation could reflect a lack of recognition by a provider. If a provider does not recognize a prediabetes diagnosis, then an opportunity to educate the Veteran about type 2 diabetes risk may also be missed.

This data provides the opportunity to bring the attention of health care providers towards prediabetes diagnosis and its documentation for at-risk Veterans. Completing diagnosis documentation is a positive step toward confirming that Veterans are aware of the condition. Recognizing the condition can help health care providers to inform Veterans of opportunities for education and lifestyle intervention to reduce the risk of progression to type 2 diabetes.

## Figures and Tables

**Table 1:** Demographics by prediabetes diagnosis.

	No Documented Code (N = 72,565)	Documented Code (N = 33,172)	Total (N = 105,737)	*p*-Value
**Age**, mean (SD)	61.3 (15.1)	61.4 (13.5)	61.3 (14.7)	0.0748 ^1^
**Gender**, n (%)				<0.0001 ^2^
Male	61,506 (84.8%)	26,983 (81.3%)	88,489 (83.7%)	
Female	11,059 (15.2%)	6189 (18.7%)	17,248 (16.3%)	
**Race**, n (%)				<0.0001 ^2^
White	39,405 (54.3%)	15,828 (47.7%)	55,233 (52.2%)	
Black	27,895 (38.4%)	15,109 (45.5%)	43,004 (40.7%)	
Asian	822 (1.1%)	409 (1.2%)	1231 (1.2%)	
AIAN	707 (1.0%)	225 (0.7%)	932 (0.9%)	
NHPI	513 (0.7%)	224 (0.7%)	737 (0.7%)	
Declined	2407 (3.3%)	1024 (3.1%)	3431 (3.2%)	
Unknown	816 (1.1%)	353 (1.1%)	1169 (1.1%)	

^1^ Kruskal–Wallis *p*-value; ^2^ Chi-Square *p*-value. Demographic characteristics by documented prediabetes code, defined as ICD-10 code R73.xx with a qualifying HbA1c value. AIAN = American Indian or Alaskan Native; NHPI = Native Hawaiian or Pacific Islander.

**Table 2:** Medications prescribed by prediabetes diagnosis.

	No Documented Code (N = 72,565)	Documented Code (N = 33,172)	Total (N = 105,737)	*p*-Value
**Antipsychotics**, n (%)				<0.0001 ^1^
Yes	4859 (6.7%)	1949 (5.9%)	6808 (6.4%)	
No	67,706 (93.3%)	31,223 (94.1%)	98,929 (93.6%)	
**GLP-1**, n (%)				<0.0001 ^1^
Yes	1081 (1.5%)	1474 (4.4%)	2555 (2.4%)	
No	71,484 (98.5%)	31,698 (95.6%)	103,182 (97.6%)	
**Immunosuppressants**, n (%)				0.3664 ^1^
Yes	497 (0.7%)	211 (0.6%)	708 (0.7%)	
No	72,068 (99.3%)	32,961 (99.4%)	105,029 (99.3%)	
**Metformin**, n (%)				<0.0001 ^1^
Yes	2739 (3.8%)	3217 (9.7%)	5956 (5.6%)	
No	69,826 (96.2%)	29,955 (90.3%)	99,781 (94.4%)	
**Prednisone**, n (%)				0.6306 ^1^
Yes	437 (0.6%)	208 (0.6%)	645 (0.6%)	
No	72,128 (99.4%)	32,964 (99.4%)	105,092 (99.4%)	
**Weight loss medications**, n (%)				<0.0001 ^1^
Yes	22,314 (30.8%)	10,713 (32.3%)	33,027 (31.2%)	
No	50,251 (69.2%)	22,459 (67.7%)	72,710 (68.8%)	

^1^ Chi-Square *p*-value. Prescription of listed medication classes by prediabetes ICD-10 code (R73.xx) with qualifying HbA1c. Medication classes are defined by VA drug classifications: antipsychotics (CN701, CN709), GLP-1 agonists (HS509), immunosuppressants (IM599, IM600), metformin (HS502), >90 day prescription for prednisone (HS051), and weight loss medications (GA751, GA900).

**Table 3:** Concurrent medical conditions by prediabetes diagnosis.

	No Documented Code (N = 72,565)	Documented Code (N = 33,172)	Total (N = 105,737)	*p*-Value
**Lipid disorders**, n (%)				<0.0001 ^1^
Yes	35,586 (49.0%)	22,168 (66.8%)	57,754 (54.6%)	
No	36,979 (51.0%)	11,004 (33.2%)	47,983 (45.4%)	
**Schizophrenia**, n (%)				0.0008 ^1^
Yes	532 (0.7%)	183 (0.6%)	715 (0.7%)	
No	72,033 (99.3%)	32,989 (99.4%)	105,022 (99.3%)	
**Depressive episode**, n (%)				<0.0001 ^1^
Yes	13,275 (18.3%)	6596 (19.9%)	19,871 (18.8%)	
No	59,290 (81.7%)	26,576 (80.1%)	85,866 (81.2%)	
**MDD**, n (%)				0.0157 ^1^
Yes	13,075 (18.0%)	6182 (18.6%)	19,257 (18.2%)	
No	59,490 (82.0%)	26,990 (81.4%)	86,480 (81.8%)	
**Anxiety disorders**, n (%)				<0.0001 ^1^
Yes	16,839 (23.2%)	8147 (24.6%)	24,986 (23.6%)	
No	55,726 (76.8%)	25,025 (75.4%)	80,751 (76.4%)	
**Stress disorders**, n (%)				0.0004 ^1^
Yes	25,465 (35.1%)	12,014 (36.2%)	37,479 (35.4%)	
No	47,100 (64.9%)	21,158 (63.8%)	68,258 (64.6%)	
**Hypertension**, n (%)				<0.0001 ^1^
Yes	38,643 (53.3%)	19,749 (59.5%)	58,392 (55.2%)	
No	33,922 (46.7%)	13,423 (40.5%)	47,345 (44.8%)	
**Heart failure**, n (%)				0.1214 ^1^
Yes	3161 (4.4%)	1376 (4.1%)	4537 (4.3%)	
No	69,404 (95.6%)	31,796 (95.9%)	101,200 (95.7%)	

^1^ Chi-Square *p*-value. Presence of comorbid ICD-10 by prediabetes documentation (R73.xx) with a qualifying HbA1c value. Comorbidities are defined by ICD-10 codes: Lipid disorders (E78.xx), schizophrenia (F20.xx), depressive episodes (F32.xx), major depressive disorder (F33.xx), anxiety disorders (F41.xx), stress and adjustment disorders (F43.xx), primary hypertension (I10.xx), and heart failure (I50.xx). MDD = Major depressive disorder.

**Table 4:** Documentation of Prediabetes Diagnosis by Facility Complexity.

	1a (N = 54,446)	1b (N = 10,319)	1c (N = 40,972)	Total (N = 105,737)	*p*-Value
**Prediabetes**, n (%)	16,898 (31.0%)	2964 (28.7%)	13,310 (32.5%)	33,172 (31.4%)	<0.0001 ^1^

^1^ Chi-Square *p*-value. Patients with a documented diagnosis of prediabetes (R73.xx) in the last 24 months with a qualifying HbA1c value by VA facility complexity. Facility complexity, defined by patient volume, patient risk level, and capacity for clinical and teaching programs, increases from 1c to 1a.

**Table 5:** Documentation of prediabetes diagnosis by rurality.

	Urban (N = 66,043)	Rural (N = 38,042)	Highly Rural (N = 1149)	Island or Not Classified (N = 503)	Total (N = 105,737)	*p*-Value
**Prediabetes,** n (%)	21,565 (32.7%)	11,068 (29.1%)	411 (35.8%)	128 (25.4%)	33,172 (31.4%)	<0.0001 ^1^

^1^ Chi-Square *p*-value. Patients with a documented diagnosis of prediabetes (R73.xx) in the last 24 months with a qualifying HbA1c value by VA GISURH classifications derived from RUCA (Rural-Urban Commuting Area) codes.

## Data Availability

The data sets presented in this article are not readily available because they are restricted to the Veterans Health Administration.

## Abbreviations

The following abbreviations are used in this manuscript:

VHA	Veterans Health Administration
VISN	Veterans Integrated Service Network
HbA1c	Hemoglobin A1c
